# Towards a framework to assess, compare and develop monitoring and evaluation of climate change adaptation in Europe

**DOI:** 10.1007/s11027-015-9678-4

**Published:** 2015-08-15

**Authors:** Judith Klostermann, Kaj van de Sandt, Mike Harley, Mikael Hildén, Timo Leiter, Jelle van Minnen, Nico Pieterse, Leendert van Bree

**Affiliations:** 1 Wageningen University and Research Centre, WUR-Alterra, P.O. Box 47, 6700 AA Wageningen, Netherlands; 2 Netherlands Environmental Assessment Agency PBL, Wageningen, Netherlands; 3 Climate Resilience Ltd, Rutland, UK; 4 Finnish Environment Institute, Helsinki, Finland; 5 Competence Centre for Climate Change, Deutsche Gesellschaft für Internationale Zusammenarbeit (GIZ) GmbH, Rabat, Morocco

**Keywords:** Climate change, Adaptation, Framework, Monitoring, National Adaptation Strategies

## Abstract

Adaptation is increasingly recognised as essential when dealing with the adverse impacts of climate change on societies, economies and the environment. However, there is insufficient information about the effectiveness of adaption policies, measures and actions. For this reason, the establishment of monitoring programmes is considered to be necessary. Such programmes can contribute to knowledge, learning and data to support adaptation governance. In the European Union (EU), member states are encouraged to develop National Adaptation Strategies (NASs). The NASs developed so far vary widely because of differing views, approaches and policies. A number of member states have progressed to monitoring and evaluating the implementation of their NAS. It is possible to identify key elements in these monitoring programmes that can inform the wider policy learning process. In this paper, four generic building blocks for creating a monitoring and evaluation programme are proposed: (1) definition of the system of interest, (2) selection of a set of indicators, (3) identification of the organisations responsible for monitoring and (4) definition of monitoring and evaluation procedures. The monitoring programmes for NAS in three member states—Finland, the UK and Germany—were analysed to show how these elements have been used in practice, taking into account their specific contexts. It is asserted that the provision of a common framework incorporating these elements will help other member states and organisations within them in setting up and improving their adaptation monitoring programmes.

## Introduction

The fifth assessment report of the Intergovernmental Panel on Climate Change (IPCC) has confirmed that it is no longer a question of if, but rather how fast, the climate is changing (IPCC et al. 2014). Convincing arguments for the need to adapt to inevitable impacts have been made to governments in Europe and globally (European Commission 2009; World 2012). Adaptation to climate change involves a broad range of measures directed at reducing economic, environmental and social vulnerability to climatic factors (Füssel 2007; Walker et al. 2013). Adaptation has also been seen to be highly context-specific, because it depends on the climatic, environmental, social and political conditions in a target location or sector (Füssel 2007).

Member states of the European Union (EU) are encouraged to develop National Adaptation Strategies (NASs) (European Commission 2013). Many member states have formulated their NAS, and there is significant progress in establishing institutional arrangements to implement them (EEA 2014). Concrete adaptation actions are beginning to emerge (Ford et al. 2011; EEA 2013). For example, England’s heat wave plan is an adaptation measure that provides guidelines for local governments and the National Health Service on how to respond to heat-related health problems (see https://www.gov.uk/government/publications/heatwave-plan-for-england-2013). In some cases, actions initially designed to serve other objectives have gained significance in relation to climate change. An example is the Dutch ‘sand engine’, a coastal protection measure which involves large amounts of sand being dumped on the seaward side of the Dutch coast and then redistributed by natural sedimentary processes (see http://www.dezandmotor.nl/en-GB/the-sand-motor/).

With the development of adaptation strategies comes the need for methods to monitor and evaluate the level of implementation and the effectiveness of adaptation policies, measures and actions. Monitoring is the systematic collection of data, based on pre-defined indicators, to enable stakeholders to check whether a policy process, programme or project is on-track and whether the stated objectives can be achieved (Lamhauge et al. 2012). The purpose of monitoring and evaluating adaptation is to follow progress in implementing adaptation policies, measures and actions, to assess the effectiveness of resource commitments and to share information on good practice (Harley et al. 2008). Systematic monitoring can also provide governance information that is required by the United Nations Framework Convention on Climate Change (UNFCCC) (United Nations 1998: Art. 10) and by the European Commission in relation to the Monitoring Mechanism Regulation (MMR (EU) 525/2013: Art. 15). Feedback from monitoring and evaluation is expected to improve adaptation policies, measures and actions. For example, systematic data collection around new adaptation solutions can show which of these are efficient and effective. Knowledge transfer and learning can also be useful to countries in the early stages of programme development.

So far, few NAS developed by EU member states have been accompanied by monitoring programmes (Biesbroek et al. 2010), although the number is increasing (EEA 2014). Finland, the UK, Germany and France are among those that have adopted approaches to monitor and evaluate their NAS (Ministry of Agriculture and Forestry of Finland 2009, 2014; Adaptation Sub-Committee 2010, 2011, 2012, 2013; UBA 2010; GIZ 2013d). Since climate adaptation is in the early stages of development, there is currently no common standard for adaptation monitoring. In contrast to the detailed requirements for reporting on actions in climate change mitigation, the Monitoring Mechanism Regulation (MMR (EU) 525/2013) gives only general guidance:

Member states shall report to the Commission information on their national adaptation planning and strategies, outlining their implemented or planned actions to facilitate adaptation to climate change. That information shall include the main objectives and the climate change impact category addressed, such as flooding, sea level rise, extreme temperatures, droughts and other extreme weather events.

The aim of this study is to support joint learning about adaptation monitoring in Europe. Radelli (2009) distinguished between social learning that involves large-scale paradigmatic changes, reflexive learning about governance, instrumental learning about what seems to work, political learning about playing the game, and cross-national emulation in which models and solutions are imported. Here, the focus is on reflexive and instrumental learning. The main research question focusses on how adaptation can be monitored at the national level to detect a possible mismatch between policy and implementation (Ford et al. 2011). Three sub-questions are posed: (1) What general requirements apply to monitoring adaptation? (2) How is adaptation monitoring different from other monitoring approaches and does this lead to additional requirements? (3) What common elements can be identified that should be included in a framework for monitoring adaptation?

This paper begins with a literature review to ascertain what the specific requirements for monitoring adaptation might be. The key elements that guide the monitoring of adaptation policies, measures and actions are then identified and examined in relation to how they have been shaped in existing programmes in Europe. Finally, suggestions are made as to how these elements could form part of a generic framework for use by member states in monitoring and evaluating their NAS.

## Method

An iterative research method, with three steps, was used to build and test a framework for monitoring and evaluating adaptation.

The first step was a critical review of the monitoring and evaluation literature, the aim of which was to identify both the general methodological requirements and the specific challenges for monitoring adaptation. A number of search terms were used to source relevant materials, including the following: monitor (climate change) adaptation, monitoring (climate change) adaptation, evaluation (climate change) adaptation, evaluate (climate change) adaptation, (climate change) adaptation indicators, and adaptive monitoring.

Despite monitoring being essentially a location or sector-specific activity, the challenge here was to streamline different approaches into a common framework that could help in the development of new programmes, or the evaluation and enhancement of existing ones. Therefore, the next step was to create an outline of a framework and its building blocks. The adaptation literature cites various approaches to establishing such a framework and the necessary elements that should be included.

The third step was to assess three NAS monitoring programmes in order to test and modify the proposed framework. Monitoring programmes from Finland, the UK and Germany were chosen because they are among the first EU member states to monitor their NAS and because their monitoring reports are publicly available. Policy documents and documents describing their strategies and monitoring programmes were analysed in accord with the set of questions detailed in Table 1. These analyses provided additional information on the content of each building block and were used to guide the development of the final framework.

**Table 1:** Questions, based on the four framework building blocks, used to analyse the NAS of Finland, the UK and Germany

Building block	Questions
System of interest	1. Is the description of the adaptation context based on a transparent and structured
	overview of:
	a. Current and future climate (preferably on the basis of downscaled climate
	models)?
	b. Important climate impacts on socio-economic and environmental systems,
	including exposure and sensitivity?
	c. Socio-economic and environmental vulnerabilities?
	d. Adaptation policies, measures and actions and their expected outcomes?
	2. Is there a definition of relevant temporal and spatial scales?
Indicators	3. What indicators are selected for monitoring and evaluation:
	a. Process indicators?
	b. Output indicators?
	c. Outcome indicators?
	4. Are indicators of the social system included, for example, for adaptive capacity?
Responsible organisation	5. Which organisation(s) is/are chosen or created to monitor and evaluate adaptation?
	6. Is the organisation dependent on or independent of the organisation responsible for
	implementing adaptation? ‘Dependent’ and ‘independent’ are here defined in an
	administrative-hierarchical sense.
	7. What financial and other resources are available to the organisation for monitoring?
	8. What are the arrangements that provide legitimacy and credibility to the organisation?
Procedures	9. Are information needs and monitoring objectives clearly described?
	10. Are monitoring procedures clearly specified, including data collection and reporting?
	11. Does the monitoring procedure enhance mainstreaming of adaptation?
	12. Do the procedures prescribe stakeholder involvement and, if so, where in the
	monitoring process?
	13. Is the notion of adaptive monitoring incorporated?

## Challenges and requirements for adaptation monitoring

As with all monitoring and evaluation processes, monitoring and evaluation of adaptation should meet a number of general requirements. Useful information has to be produced based on credible and legitimate indicators (Walker et al. 2013). These indicators should be precise, robust, transparent, objective, simple and easy to understand, and they should be linked to appropriate datasets (Harley et al. 2008; Spearman and McGray 2011). Furthermore, indicators should meet SMART criteria: specific (target a specific area for improvement), measurable (quantify or at least suggest an indicator of progress), assignable (specify who will do it), realistic (state what results can realistically be achieved, given available resources), and time-related (specify when the result(s) can be achieved) (Doran 1981; Glahn et al. 2007). In order to assure the credibility of its outputs, the monitoring organisation should ideally have an independent status (UNDP Evaluation Office 2002), especially where the evaluation is to be communicated publically.

In addition to these general requirements, adaptation monitoring should respond to a number of specific challenges. Below, several of these challenges are identified from the literature, and their consequences for monitoring and evaluation are discussed.

First, adaptation is characterised by different aspects of uncertainty, especially where a policy, measure or action is intended to anticipate future changes (Wilby and Dessai 2010). There is uncertainty about the magnitude of climatic changes and about the changes in probability of extreme events (IPCC 2012). The uncertainties and potential surprises implicit in planning for multi-decadal climate change are complicated by the fact that society may also undergo fundamental economic and cultural changes (Hallegatte 2009; Bours et al. 2014). The effectiveness of adaptation policies, measures and actions may only become measurable in the future (Brooks et al. 2011). To tackle this challenge, the concept of adaptive management provides a basis from which to improve adaptation (Harley et al. 2008). First introduced in the field of ecology (Holling 1978), adaptive management can be defined as a systematic learning process that works in parallel with the implementation of policies and practices (Pahl-Wostl et al. 2007). Continuous monitoring plays an essential role in adaptive management (Wilby and Dessai 2010; Ford et al. 2011). It promotes learning and the ability of decision-makers to respond to social and environmental change (Cundill and Fabricius 2009). Monitoring of adaptation itself also needs to be flexible, because new knowledge about adaptation policies, measures and actions will become available, and also because the way in which society frames adaptation may change (Howden et al. 2007; Bours et al. 2014). Therefore, adaptive monitoring requires detailed decision procedures that should underpin the monitoring programme. These procedures must foster contacts between scientists and policy makers who discuss how indicators connect adaptive monitoring with adaptive management (Lindenmayer et al. 2011).

Second, shifting baselines mean that evaluation is likely to take place against the backdrop of a changing norm (Harley et al. 2008; Bours et al. 2014). This suggests that an increase in a climate-related impact as the climate changes may prevent an adaptation policy, measure or action from being successful (Brooks et al. 2011). To address this challenge, the climate itself should be monitored to enable adaptation policies, measures and actions to be normalised against a shifting baseline (Harley et al. 2008; Wilby and Dessai 2010). Examples of climate indicators include changes in temperature and precipitation, and in the frequency of extreme events such as floods and droughts (Goosen et al. 2013).

Third, monitoring of adaptation should address the issue of attribution. Attribution seeks to identify the factors, in addition to adaptation policies, measures and actions, which may shape adaptation outcomes (UKCIP 2011; Bours et al. 2014). Attribution challenges occur in all areas of monitoring but can be more problematic in adaptation due to long timescales and the implementation of measures and actions across a number of policy areas and/or sectors (mainstreaming). However, mainstreaming has advantages in that existing indicators used in policy areas and sectors may have relevance to adaptation and be able to link measures and actions back to the NAS. Problems associated with attribution can be reduced through a clear description of the system(s) being monitored and through hypotheses or causal chains outlining how policies, measures and actions are likely to contribute to an intended objective (GIZ 2013a). This allows the contribution of a policy, measure or action to a certain outcome to be plausibly stated.

Fourth, adaptation takes place in a multi-stakeholder environment. It has a diverse, multi-sectoral nature and involves a large number of responsible organisations (Gardner et al. 2009; Sherman and Ford 2014; Bours et al. 2014). Monitoring objectives differ between stakeholders in line with their responsibilities in the adaptation process and their discursive frames. A discursive frame comprises the cultural viewpoints used by a community or an organisation to make sense of reality and to decide upon their practices (Benford and Snow 2000). For example, the EC might wish to compare adaptation strategies across Europe, while individual member states might be more interested in the efficiency of their adaptation policies, measures and actions. Differences in adaptation strategies and monitoring preferences between member states are likely due to differing vulnerabilities and governmental traditions (Swart et al. 2009; Brännlund 2010). At local scales, within regions and municipalities, stakeholder groups will have different perceptions and information needs, depending on the adaptation context—a combination of the physical and socio-economic setting, and the adaptation objectives (Füssel 2007).

In summary, effective monitoring and evaluation programmes for adaptation should meet the general requirements of monitoring: credibility, policy relevance and high-quality methods. They should also address the specific challenges of adaptation: uncertainties and shifting baselines, and attribution and the multi-stakeholder context. There is no one-size-fits-all approach to adaptation: responses need to be tailored to specific circumstances (UKCIP 2011; GIZ 2013b). Likewise, monitoring programmes should be tailored to the adaptation context and to information needs (van Minnen et al. 2014). This implies that stakeholders must be involved in the monitoring and evaluation process (Swart et al. 2009). Agreement must be sought on the focus, aims and goals of the monitoring, on the use of indicators and on the governance structure (van Minnen et al. 2014; Leiter 2013). Table 2 provides a summary of the general requirements and challenges and links these to potential solutions.

**Table 2:** Overview of general challenges for monitoring and specific challenges for adaptation monitoring.

General challenges for monitoring	Proposed solutions
Useful information: salient and context sensitive, responsive to specific information demands	Involve stakeholders to check information needs Research mechanisms in system(s) of interest
Technical quality of indicators: accurate, valid, precise, robust, meet SMART criteria	Use/develop review procedures Use existing indicators/data sources Research physical mechanisms in system(s) of interest
Communicative value and efficiency of indicators: simple and straightforward to understand	Test communicative value of indicators Use existing well-known indicators
Credible production of information: unbiased, legitimate, transparent, objective/independent	Scientifically sound methods Independent operation of monitoring organisation
Monitoring must be feasible: availability of data, limited financial and human resources	Limit the set of indicators Use existing datasets Evaluate usefulness of indicators
Specific challenges for adaptation monitoring	Proposed solutions
Coping with uncertainties	Adaptive monitoring Design for learning
Addressing shifting baselines	Monitor background variables for climate and economy
Demonstrating contribution (we use of contribution rather than attribution, acknowledging that an outcome is a combined effect of several factors; see Bours et al. 2014, point 9)	Use theories of change to describe causal mechanisms Combine qualitative, quantitative and binary indicators Create links with adaptation measures
Meeting stakeholder needs	Involve stakeholders in the monitoring and evaluation process

## Building blocks for a monitoring framework

Although monitoring programmes should be tailored to specific locations or sectors, there are common elements that can be combined to create a generic framework. This should help stakeholders develop new monitoring programmes, or evaluate or improve existing ones.

### Key elements of existing frameworks

The adaptation literature cites various approaches to monitoring and evaluation and the necessary elements that should be included. Lamhauge et al. (2012), for example, analysed 106 documents produced by development organisations to evaluate adaptation. These authors found that Result Based Management (RBM) and the Logical Framework Approach (LFA) are most commonly used for monitoring and evaluating adaptation programmes and projects. RBM is defined as a management strategy focusing on performance and achievement of outputs, outcomes and impacts (Lamhauge et al. 2012, after OECD 1993). LFA (or logframe) follows a logical hierarchy of objectives: Activities deliver outputs, which contribute to outcomes, which help bring about the overall goal. It assesses progress against each objective with indicators, means of verification and external factors such as assumptions and risks (Bakewell and Garbutt 2005). A related approach is the Theory of Change (TC), which is also based on the idea of causal relationships between contextual factors, actions and outcomes; however, this approach is more fuzzy as it does not assume that everything about a system is known or will be known (Retolaza Eguren 2011).

Adaptation indicators can be sub-divided into process, output and outcome-based indicators (e.g. Harley et al. 2008; Swart et al. 2009; Harley and van Minnen 2009; Cundill and Fabricius 2009; UKCIP 2011; Bours et al. 2013). Process-based indicators monitor the policy, institutional and governance processes needed to build capacities to develop and implement adaptation policies, measures and actions. Output-based indicators capture the implementation of adaptation policies. They show that governments and other stakeholders have kept word, but they do not show if vulnerability has been reduced. Output-based indicators include mainstreaming with other (existing) environmental and/or sectoral policies (Ahmad 2009). Outcome-based indicators measure the effectiveness of adaptation policies, measures and actions (see also Spittlehouse and Stewart 2003; Spearman and McGray 2011), including the adaptive capacity of people, institutions and governance structures in relation to the delivery of adaptation policies, measures and actions (Georgi et al. 2012). Process-based and output-based indicators are easier to establish initially, and once policy goals become more targeted, outcome-based indicators will assume a greater importance (Harley et al. 2008; Harley and van Minnen 2009).

Brooks et al. (2011) produced a framework for monitoring adaptation from a development perspective. The framework comprises process-based indicators to monitor “the capacity of institutions, government and civil society to understand climate change and to integrate adaptation into decision-making”, and outcome-based indicators to monitor “the extent to which climate adaptation keeps development ‘on track’ in the short term”. Processes are monitored from a top-down perspective and outcomes (performance) from a bottom-up perspective.

The capacity of institutions and society to adapt to climate change is determined by a range of factors, such as good governance, competences and commitment (Georgi et al. 2012). These factors can be grouped under three headings—awareness, ability and action—which may be relevant to monitoring adaptive capacity.

Bours et al. (2013) published an overview of the tools, frameworks and approaches used in monitoring and evaluating climate change adaptation. In their conclusion, they advocate a conceptual shift beyond the mere purpose of accountability, to a more critical and creative process of knowledge exchange. In this way, monitoring and evaluation will become a tool for improvement and learning, and not simply a reporting mechanism.

In its guidelines, GIZ (2013c) outlines a four-step generic approach to assist countries in setting up an adaptation monitoring programme: (i) analysing the context and defining objectives, (ii) choosing an appropriate methodology, (iii) formulating indicators and (iv) operationalising the monitoring. Leiter (2013) provides good practice examples from a number of pioneering countries. This is complemented by a comparative analysis of 10 national adaptation monitoring and evaluation systems, which considers three components: (i) the context of the monitoring and evaluation system, including its objectives, the relationship with national policies and the scale of implementation; (ii) the monitoring and evaluation process, including institutional arrangements and the development process; and (iii) the content of the monitoring and evaluation system, including the methodology and indicators used, as well as the information outputs and required resources (GIZ 2013d). The analysis points to the diversity of national systems for monitoring and evaluating adaptation and demonstrates the potential for learning from these pioneering approaches.

The Global Programme of Research on Climate Change Vulnerability, Impacts and Adaptation (PROVIA 2013) produced guidance on assessing impacts, vulnerability and adaptation to climate change for use at international, national and local levels. The conceptual basis, decision trees and methods and tools included in the guidance build on research conducted within the Methodology for Effective Decision-making on Impacts and Adaptation project (MEDIATION 2013). PROVIA updated and improved existing guidelines and discussed key issues at each stage of the adaptation learning cycle. The cycle comprises the following phases: (1) identifying adaptation needs, (2) identifying adaptation options, (3) appraising adaptation options, (4) planning and implementing adaptation options and (5) monitoring and evaluation of adaptation. The aim of the fifth step is to identify any problems, document the outcomes achieved, change course as needed and draw lessons from the experience. The learning derived from monitoring and evaluation should also be beneficial to the adaptation process itself, which may require refinement through iterations with end-users.

### A proposed common framework for monitoring and evaluating adaptation

**Fig. 1:**
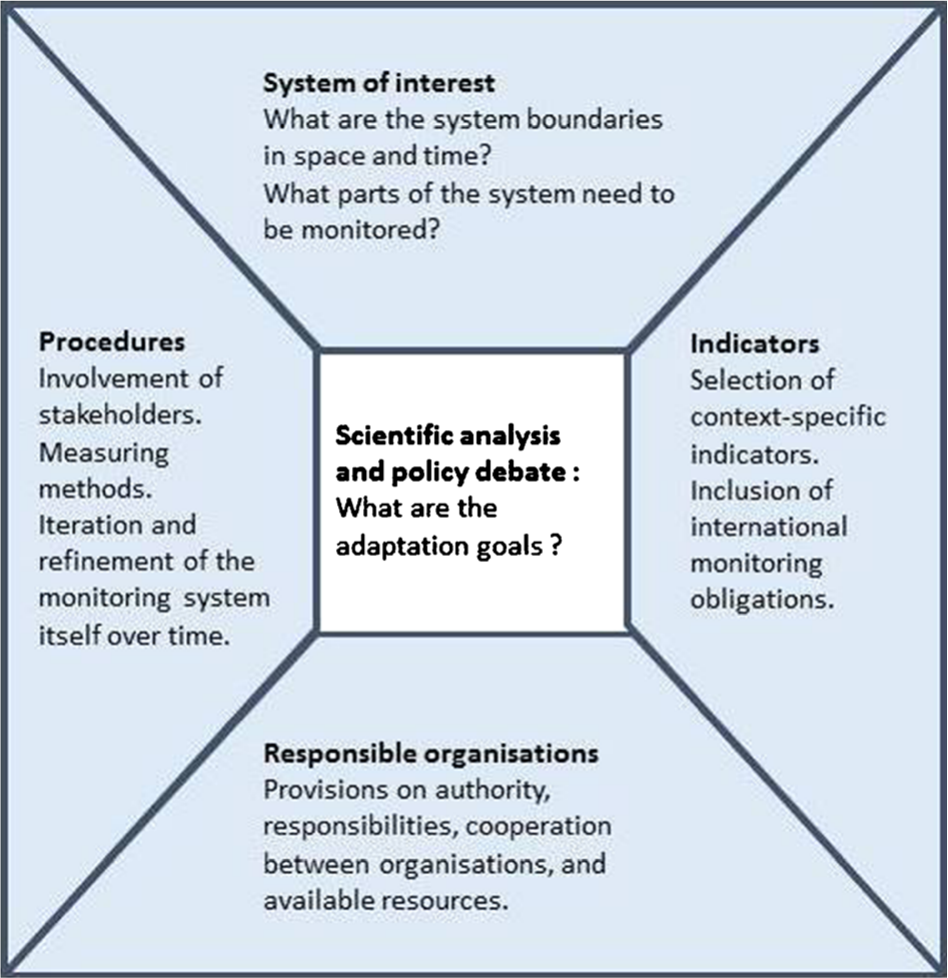
Outline of a framework for monitoring adaptation

The review and assessment of relevant literature, coupled with reflective iterations, enables us to define the elements of a common framework for monitoring and evaluating adaptation. The framework consists of four building blocks: (1) definition of the system of interest, (2) selection of the indicator set, (3) identification of the organisations responsible for monitoring and (4) definition of monitoring and evaluation procedures. The individual building blocks are described below. Figure 1 shows the framework diagram. The starting point is a scientific and policy-relevant debate to identify the adaptation policies, measures and actions. Once these are clear, the framework can guide users to describe the system of interest, and to identify and select the most appropriate indicators. The monitoring process involves iteratively revisiting the four building blocks. The framework will need to be adapted to specific contexts in order to be of value in supporting the development of adaptation monitoring programmes.

#### Definition of the system of interest

The system of interest defines which parts of the physical and social context are to be monitored. In practice, these are likely to be found in adaptation strategies and associated objectives. Such strategies will describe the expected impacts of climate change on specific aspects of society and the environment (e.g. a river basin) and will often suggest adaptation solutions. Systems of interest include specific sectors (e.g. agriculture and water management), specific functions (e.g. transport and energy production) and geographically specific areas (e.g. mountains, coastal zones). Relevant information on the system of interest may also be found in background documents, such as climate change impact and vulnerability assessments.

The adaptation context is often described through the concepts of impacts, vulnerability, resilience and adaptive capacity in relation to a particular system of interest. These concepts are widely used in the natural and social sciences, often with different meanings (Gallopín 2006). This research uses the conceptual model developed by Füssel and Klein (2006), which links the climate system, climate impacts, adaptive capacities, vulnerabilities and adaptation measures, and provides information on exposure, sensitivity, potential impacts, adaptive capacity, vulnerability and adaptation action. The socio-political situation and climatic drivers also form a part of this description.

Local research is needed to better understand the mechanisms operating within the system of interest (e.g. to obtain better insights into causal relationships, the links between measures and intended outcomes, and how these might be visualised). This will be important in determining precisely what needs to be monitored within the system of interest, and how and when monitoring should be undertaken in the adaptation process. It should also lead to a more effective engagement of and communication with a range of stakeholders.

#### Selection of a set of indicators

The selection of indicators will be determined by the prevailing adaptation context in the system of interest. In each governance context, the challenge will be to prioritise, combine or aggregate indicators so that they give an overarching picture of preparedness (van Minnen et al. 2014). The selection of a robust subset of variables on which to focus is a key consideration in the development of these indicators (Füssel 2007).

It is recognised that indicators are not without their limitations (Hinkel 2011); These relate particularly to issues of uncertainty, shifting baselines and attribution that challenge the integrity of the knowledge required to construct valid indicators. To address this challenge, the framework is founded on the principles of adaptive management, with initial indicator selection being revisited iteratively in each learning cycle and revised accordingly.

The literature review provides some basic advice for the selection of indicators (see also Table 2): Use existing indicators and/or data sources, if possible; monitor changes in climate and socio-economic variables to enable data to be normalised; combine qualitative, quantitative and binary indicators; and create explicit links with adaptation measures (GIZ 2013a).

It is likely that a combination of process-based, output-based and outcome-based indicators will be needed to monitor progress in adaptation. Process-based indicators can be used to describe the steps and decisions taken in the adaptation process (Table 3). Output-based indicators can show the level of implementation of adaptation policies. Outcome-based indicators measure the effectiveness of adaptation actions in reducing the vulnerability of the system of interest. Distinguishing between these categories is useful when building a balanced set of indicators. However, in reality, drawing a line between the different categories can be difficult; because of complex chains of events, differentiating between what is a process and what is an output, for example, can be arbitrary.

**Table 3:** Types of adaptation indicators (based on Harley and van Minnen 2009 and Mickwitz et al. 2009)

Process-based indicators	
Aspect	Description
Development	Development of adaptation policies, measures and actions
Implementation	Steps in the process of implementing adaptation measures and actions
Output-based indicators	
Aspect	Description
Inclusion	Extent to which climate change policy objectives and adaptation impacts have been covered
Consistency	Efforts to minimise contradictions between climate change adaptation and mitigation and other policy goals
Weighting	Determine relative priorities of climate change adaptation compared to other policy aims
Reporting	Evaluation and reporting requirements for climate change adaptation (including deadlines)
Resources	Availability of economic and other resources (including knowledge)
Outcome-based indicators	
Aspect	Description
Awareness dimension	Public access to information about climate change, perception of risks, and human and social capital
Ability dimension	Potential of society to design and implement adaptation measures
Action dimension	Implementation and effectiveness of adaptation solutions

#### Identification of the organisations responsible for monitoring

Effective and efficient monitoring requires a clear definition of the responsibilities for data gathering and evaluation (Biesbroek et al. 2010). Adaptation is an interdisciplinary exercise, which takes place in a multi-stakeholder environment and, therefore, the necessary institutional arrangements can be complex. Each area to be covered must be coordinated by a trusted organisation that can provide staff of sufficient (scientific) quality, with the gathered data being both credible and legitimate. A responsible organisation should be permanent and equipped with appropriate resources, as it will need to gather data on climate adaptation on an ongoing basis (Swart et al. 2009). A general coordinator, with the task of maintaining an overview of all the monitoring activities, can help in synthesising the information.

Organisational independency may be desirable if the main objective of monitoring and evaluation is to account for adaptation policies, measures and actions. In such cases, an independent commission could be created to report on adaptation at a high political level (e.g. directly to a parliament). However, monitoring programmes with a strong learning focus may need a different approach, with more dependent organisational relationships (e.g. a temporary internal team with a broadly formulated mandate).

#### Definition of monitoring and evaluation procedures

Monitoring procedures are detailed study plans that guide how data are to be collected, managed, analysed and reported (Oakley et al. 2003). Such procedures can ensure that data meet defined quality standards with a known level of confidence, that they detect changes over time and that they allow for comparisons of data among places and agencies. Methods for measuring indicators have to be transparent and scientifically sound.

Clear monitoring procedures are necessary to involve stakeholders in the monitoring and evaluation exercise, depending on their information needs. The communicative value of indicators can be tested among intended end-users, and review procedures can be developed to evaluate their usefulness in practice.

Adaptation monitoring programmes need to be flexible in order to deal with uncertainties and new insights (Howden et al. 2007; Bours et al. 2014). Adaptive monitoring incorporates active learning into monitoring programmes, preferably without distorting or breaching the integrity of the data record (Lindenmayer and Likens 2009). Adaptive management can be supported by monitoring so-called triggers for change (i.e. essential environmental aspects, such as mean sea level). As soon as a rapid change is detected in one of the triggers for change indicators, the adaptation pathway can be adjusted (Wilby and Dessai 2010).

#### Applying and learning from the framework

Figure 1 shows the different building blocks for the design of a monitoring programme. Users can choose to work through the framework in a clockwise or counter-clockwise direction. By choosing clockwise, users will opt for a science-dominated approach. After first identifying (an outline of) the system of interest, indicators will be selected to reflect the adaptation context of that system (e.g. changes in vulnerable and risk). In the next step, organisations responsible for monitoring the system will be chosen. The monitoring and evaluation procedures will then be negotiated by these organisations. The organisations may have to adapt to enable them to perform all the required functions. By choosing counter-clockwise, users will first choose a responsible organisation for creating an overview. This organisation will determine which parts of the system of interest will be monitored and will facilitate the selection of indicators and the design of procedures. Although this approach has its limitations, it can be a pragmatic way of getting a first monitoring effort started. A combination of both approaches could result in more indicators being selected and other organisations being added to the monitoring network.

The cyclical iterative approach of this framework should stimulate learning in a wide range of adaptation contexts. It implicitly promotes learning through monitoring and evaluation at all stages of the adaptation policy cycle. This is particularly important in the early stages of adaptation policy development, as learning through monitoring and from experience will provide valuable information on “are we doing the right things”. The framework is intended to be applicable across all levels of adaptation governance and to all policy areas and sectors.

## Comparing three existing monitoring programmes

Using the framework proposed above, monitoring of the NAS in three EU member states—Finland, the UK and Germany—was examined. The set of questions in Table 1 guided the analyses for each building block. The analysis for each country and an overview of the results are provided below.

### Monitoring and evaluation of Finland’s NAS

Finland was the first country in the world to adopt a NAS (Ministry of Agriculture and Forestry of Finland 2005). The NAS was prepared by a group of Finnish ministries and coordinated by the Ministry of Agriculture and Forestry. It is linked with the National Energy and Climate Strategy (Ministry of Agriculture and Forestry of Finland 2005; Ministry of Employment and the Economy of Finland 2013).

The main climate problems and adaptation solutions relating to Finland’s systems of interest are set out in the NAS (Ministry of Agriculture and Forestry of Finland 2005) and the information needed for monitoring is detailed in the NAS review (Ministry of Agriculture and Forestry of Finland 2009). The NAS begins with an in-depth description of projected climate change and then considers the impacts on 11 sectors. Current and future climate impacts are mapped, and the potential implications of these are discussed. Finally, the NAS identifies a set of possible adaptation measures. All measures (anticipatory and reactive) are described and labelled in terms of responsibility (e.g. public or private sector) and time scale (i.e. immediate, 2005–2010; short term, 2010–2030; and long term, 2030–2080). The spatial scale of the NAS is predominantly national, although finer resolution is used in some cases.

The NAS lists several existing indicators, such as those relating to water management, which were expected to provide information on progress with adaptation (Ministry of Agriculture and Forestry of Finland 2005). The level of adaptation was assessed with a policy process indicator consisting of four criteria, each on a scale of 1 to 5:

- A need for adaptation is recognized (process)
- Impacts are known (adaptive capacity)
- Adaptation measures are taken (output)
- There is cross-sectoral cooperation (mainstreaming)

The key monitoring objective was to establish what progress had been made in different sectors since the NAS was launched in 2005. A preliminary aggregated indicator for the level of adaptation, based on the four process criteria, was used as a measure of progress. This was based on a qualitative evaluation that provided only indicative information and was used mainly for self-evaluation.

The evaluation of adaptation processes was carried out by an ad hoc Coordination Group for Adaptation to Climate Change (Ministry of Agriculture and Forestry of Finland 2009). The Coordination Group was chaired by the Ministry of Agriculture and Forestry and consisted of 32 members from different ministries, research institutes, research funding agencies and regional organisations. Stakeholders from key sectors were also involved in the group. As the group was chaired by the ministry that coordinated the NAS, it cannot be considered to be independent. The group did not have separate funding; all participating members were expected to cover their own costs, with the Ministry of Agriculture and Forestry covering administrative costs and the costs of report writing.

In terms of procedures, members of the Coordination Group were responsible for assessing the level of adaptation in different sectors. Each sector was reviewed by a representative member, who produced the required information, either individually or with the assistance of other experts. The draft final report was circulated among group members for comments and discussed at group meetings, before being approved in 2009.

In 2013, the NAS was re-evaluated by a new ad hoc Coordination Group (appointed in May 2012), with sectors evaluating their own activities (e.g. Ministry of the Environment—Hildén and Mäkinen 2013). The focus was still on processes but included consideration of outcomes in specific sectors (Ministry of Agriculture and Forestry of Finland 2014). A new NAS (now called an “adaptation plan”) was approved by the Government in 2014.

### Monitoring and evaluation of the UK’s NAS

The Climate Change Act (2008) established a legislative framework for the UK to achieve its long-term goals to reduce greenhouse gas emissions and to adapt to the impacts of climate change (The National Archives 2008). Alongside the targets to reduce emissions, the Act established several duties for Government in relation to adaptation:

- To undertake a statutory Climate Change Risk Assessment (CCRA) associated with climate change impacts every five years (the first was published in 2012)
- To publish a National Adaptation Programme (NAP), based on the CCRA, every five years (the first was published in 2013)
- To establish an Adaptation Sub-Committee (ASC) of the Committee on Climate Change to advise on and scrutinise Government’s performance on adaptation

The NAP sets out Government’s policies and objectives for adaptation and addresses the risks (and opportunities) identified by the CCRA (Defra 2013). The CCRA focussed on scientific assessments of projected climate impacts and vulnerabilities for different systems of interest and sectors (Defra 2012). The NAP is structured around seven themes—built environment, infrastructure, healthy and resilient communities, agriculture and forestry, natural environment, business, and local government—and associated priority risks. To address these risks, each theme has between four and six separate adaptation objectives and each objective a number of specific actions (374 in total).

The ASC is an independent body, which is provided by Government with the human and financial resources needed to monitor adaptation. Its members and secretariat have scientific and policy backgrounds, but they do not represent stakeholder interests. The ASC has a statutory duty to report to Parliament with an independent assessment of Government’s progress in implementing its NAP.

The first report (due in 2015) will assess whether the NAP is enabling the UK’s preparedness for the climate change risks that it faces. In support of this, the ASC has published annual indicator-based reports on progress with adaptation (Adaptation Sub-Committee 2010, 2011, 2012, 2013, 2014). These documents describe the UK’s key systems of interest and assesses the preparedness of related sectors for the main risks identified by the CCRA (Adaptation Sub-Committee 2012; Adaptation Sub-Committee 2013; Adaptation Sub-Committee 2014).

The NAP identifies the need for a monitoring and evaluation framework to determine whether the programme is making a difference to the UK’s vulnerability in the near-term. The ASC has developed an adaptation assessment toolkit for this purpose. The toolkit includes an indicator framework, which uses the indicators from its progress reports (over 200) to track trends in vulnerability to/preparedness for climate change. Expert knowledge is used to augment and interpret trends identified by the indictors (which are essentially output- and outcome-based).

The ASC’s 2015 report will follow the NAP’s structure, with each theme split according to priority risks. For each risk, the ASC will

1. Monitor whether, how and why the risk is changing
2. Review implementation of relevant actions to address the risk
3. Evaluate and comment on progress towards addressing the risk

These analyses should enable the ASC to answer three key questions that relate to the strategic outcomes of the NAP:

1. Would achieving each objective help address the risks identified by the CCRA (i.e. has a tolerable level of risk been set for each objective, as well as a clear timescale over which the risk will be addressed through adaptation action)?
2. To what extent could the actions listed in the NAP help meet their associated objectives, if implemented (i.e. what is the relative contribution/importance of each action to meeting its associated objective)?
3. To what extent is implementation of these actions, and other actions, making a difference to vulnerability in the near-term?

### Monitoring and evaluation of Germany’s NAS

The German Adaptation Strategy (DAS) was adopted by the Federal Government in 2008 and is aligned with the Federal Sustainability Policy (Bundesregierung 2008). The strategy identifies 15 priority fields of action and describes climate change impacts and possible adaptation options for each of them. An Adaptation Action Plan (APA), published in 2011, specifies actions to be taken by the Federal Government (Bundesregierung 2011).

The DAS proposes the development of a methodological approach to vulnerability assessments and for indicator-based reporting to review the progress of adaptation at the Federal level. Since 2008, the Federal Environment Agency (UBA), on behalf of the Federal Ministry for the Environment (BMUB), has been coordinating a multi-stage process to establish an indicator-based monitoring system for the DAS. The UBA operates in close cooperation with the responsible ministries and is, therefore, classified as dependent.

UBA applied the DPSIR approach (driving forces, pressures, states, impacts, responses) in developing a monitoring system, the purpose of which is to provide an overview of key climate change impacts and adaptation progress for every action field at the federal level (Umweltbundesamt 2010). A six-step approach was used for the identification of indicators, including defining subject areas, generating indicator ideas in expert discussions and testing their feasibility. In a final step, the proposed list of indicators will be reviewed by government agencies for approval. Overall, some 100 indicators are expected to be chosen (GIZ 2013d).

A methodology for standardised vulnerability assessments at the federal level and respective indicator development is undertaken in a separate process. The suggested adaptation response indicators represent a mix of output and outcome-based indicators. Since they are not directly linked to particular adaptation measures or policies, additional analysis would be required to assess the effectiveness of adaptation measures. For the most part, the DAS monitoring system does not include indicators of adaptive capacity or mainstreaming. Results from the vulnerability assessment and from monitoring and evaluation of the DAS will both be used to inform the review of the DAS, which is scheduled for the end of 2015.

An extensive stakeholder consultation was undertaken to develop the monitoring system; this involved more than 260 people from relevant federal levels of government and Bundesländer, academia and the private sector (Umweltbundesamt 2011; Rotter et al. 2013). Particular emphasis was placed on utilising existing data sources and connections with existing monitoring activities at federal and Bundesländer level to facilitate implementation and reduce expenses.

### Similarities and differences in the NAS monitoring programmes

Table 4 shows a comparative overview of the NAS monitoring programmes from Finland, the UK and Germany.

**Table 4:** Overview of analyses of the NAS of Finland, the UK and Germany

Building block	Aspects	Finland	UK	Germany
Responsible organisation	Organisation	Coordination Group for Adaptation to Climate Change	Adaptation Sub-Committee	Federal Environment Agency, with existing organisations at Federal and Bundesländer level
	Dependency	Dependent	Independent	Dependent
	Resources	Provided with human resources and limited funding for synthesis work	Provided with human and financial resources	Significant resources for development of monitoring system; focus on utilising existing data sources minimises ongoing expenses.
	Legitimacy and credibility	Corresponds to normal practice in drafting strategic documents	Established by law	No information
System of interest	Climate system	In-depth analysis based on different projections	In depth analysis based on up-to-date projections (UKCP09)	In depth analysis based on four downscaled climate models
	Climate impacts	Sectoral analysis of natural and socio-economic systems; advantages and disadvantages described	Impact analysis using social, economic and environmental indicators; in-depth analysis of key impacts	Impacts analysis for some sectors
	Vulnerability	Not described in 2009; in 2013, review of vulnerabilities considered	Basic assessment of vulnerabilities in most sectors; in-depth assessment of most vulnerable sectors	Vulnerability assessments conducted in separate process at Federal level
	Adaptation measures	Identified for each sector, along with responsibilities and timing	Identified for some vulnerable sectors in ASC reports	Possible measures are identified for each action field
	Temporal scales in strategy	Clearly delineated: immediate (2005-10), short term (2010-30), long term (2030-80)	Clearly delineated: 2020s, 2050s and 2080s	No specific time frame
	Spatial scales in strategy	Mostly national level, finer resolution for some sectors	UK, country, regional, local and case study area levels	Mostly Federal level, finer resolution for some sectors
Indicators	Adaptation process	Part of aggregated indicator for level of adaptation	None	No indicators on government process
	Adaptation output	Part of aggregated indicator for level of adaptation	Emphasis on output measurement, as this best reflects government responsibilities	Focus on actions and not policy processes
	Adaptation outcome	Not identified in 2009; outcomes discussed in 2013, but not expressed in indicators	E.g. the number of households at reduced flood risk due to construction of flood defences	Response indicators describe status of adaptation (e.g. structural quality of water bodies); no quantified adaptation targets in DAS
	Adaptive capacity	Part of aggregated indicator for level of adaptation	Includes ability of institutions to deal with long-term effects	Partly reflected in some indicators, but not main focus of DAS monitoring system
Procedures	Information needs	Defined for NAS monitoring and review	Not explicitly defined; adaptation progress assessed and information for NAP development provided	To demonstrate, document and interpret climate changes and climate impacts, and monitor adaptation measures
	Data collection	Not systematic; mainly self-assessments by sectors	Data sources referred to and monitoring methods well-described	Coordination by Federal Environment Agency in collaboration with responsible government agencies; indicator factsheets specify methods of data collection, data sources and interpretation of each indicator
	Reporting	Based on sector responsibilities; indicative interval of revision 6-8 years	Legally bound to assess risk every five years	DAS will be reviewed every five years
	Stakeholder involvement	Partly engaged in Coordination Group; special events organised for evaluation	None	Stakeholder engagement in development of indicators
	Mainstreaming	Implementation of adaptation policies and measures explicitly based on sector responsibilities	Not mentioned in CCRA or ASC reports	Important aspect of DAS; Federal and Bundesländer governments are expected to integrate adaptation
	Adaptive monitoring	Not mentioned	Not explicitly mentioned, but ASC assessments executed in an adaptive manner	Adaptability is a general requirement

The three NAS and their related monitoring activities describe systems of interest in a structured and scientific way and attempt to link climate change with potential impacts at the national scale. Both the description of adaptation measures and local scale analyses are under development. A number of differences were also observed. The UK’s programme provides detailed information and a more complete picture of the vulnerability and preparedness of key systems and sectors. In Germany, national vulnerability assessments are being undertaken in parallel with monitoring the DAS (www.netzwerk-vulnerabilitaet.de). The revised Finnish NAS will address vulnerability at a general level (Ministry of Agriculture and Forestry of Finland 2014).

The indicators selected in the UK and Germany focus on the outputs and outcomes of adaptation activities, whereas those in Finland are concerned with adaptation processes. The ASC’s toolkit includes an indicator framework, which uses output and outcome-based indicators to track trends in vulnerability to and preparedness for climate change. The response indicators of the German NAS describe outcome variables for every action field but do not measure the effectiveness of adaptation actions. However, outputs and outcomes can be hard to identify at this early stage in the adaptation process and are further complicated by a range of influencing factors, of which adaptation policies, measures and actions are only a part. In Finland, a policy process indicator is used to establish adaptation progress in different sectors.

All three countries have established dedicated monitoring organisations. In Finland, the ad hoc Coordination Group is linked to the Ministry of Agriculture and Forestry, and its formation was based on a ministerial decision. The UK’s Adaptation Sub-Committee (ASC) was established under the Climate Change Act, which implies a longer-term commitment. The development of the monitoring system for the German strategy has been coordinated by the Federal Environment Agency and will be implemented in collaboration with responsible organisations at federal and Bundesländer level. The ASC has an independent position, which is supported by its legal status. In Finland and Germany, adaptation monitoring seems an internal learning effort, which is reflected in the more or less dependent status of the responsible organisations.

Adaptation monitoring procedures are most transparent in Germany. In Finland, they remain implicit within the learning process, whereas in the UK, certain legal and scientific aspects are made explicit, whilst interactions with stakeholders are not specified. Differences were also found in how monitoring activities are linked to the NAS of the three countries. In Finland, policy-makers and stakeholders were involved in the Coordination Group and the evaluation of outputs, and special stakeholder events were organised. Also, the selection of indicators is essentially a bottom-up process, with the results being used directly in adaptation planning. The UK’s ASC functions as an independent institution and is responsible for indicator selection. This is based on advice from experts and on inputs from governmental organisations with responsibility for various aspects of the NAS. In Germany, the Federal Environment Agency, which advised the Ministry for the Environment in establishing the NAS, was also given the task of coordinating the development of its monitoring system. Indicators were developed separately for each action field, based on scientific criteria and discussion with stakeholders (Rotter et al. 2013). The shortlist of indicators will be reviewed by respective government organisations prior to approval.

## Discussion and conclusions

The generic framework proposed in this paper is designed to help countries identify how they might shape the monitoring and evaluation of their adaptation efforts. The building blocks highlight the framing of the systems of interest, the variables to be monitored, the key organisations to be involved and relevant procedures to be put in place (Fig. 1). The framework supports reflexive and instrumental learning (Radelli 2009). Analyses of the governance of existing programmes, based on the building blocks of the framework, show both similarities and some significant differences (Table 4). For example, Finland’s focus on learning and the UK’s focus on accountability relate to monitoring being tailored to meet country-specific needs. Data collection varies from improvised self-assessment to strictly controlled systematic monitoring. The extensive involvement of stakeholders and a focus on procedures characterise the German programme. These differences demonstrate the need for flexibility when applying the framework.

Adaptation indicators alone cannot show why adaptation does or does not work, as this can only be explored in retrospect with evaluations based on monitoring results. The three NAS analyses have highlighted the challenges in developing adaptation indicators. From these, it is clear that a combined focus on processes, outputs and outcomes is needed to reveal the complex pathways that turn national policy into effective action (Ford et al. 2011; EEA 2014).

The building blocks of the proposed framework are generic and can be applied to both climate and non-climate adaptation processes. However, it is important to determine how the monitoring of climate adaptation differs from other monitoring exercises at the strategic level and whether the differences lead to additional demands on the monitoring. It is also important to recognise that climate adaptation involves many spatial scales. While actions on the ground are local and context-specific, this does not mean that the implementation of adaptation strategies is determined only by local action. Therefore, the system of interest (Fig. 1) and its variations are particularly important in adaptation strategies.

The analyses of the NAS and related policy documents found much information describing the system(s) of interest (building block 1) and indicators used (building block 2). However, details of monitoring organisations (building block 3) were more difficult to identify, and published reports often contained even less information about monitoring procedures (building block 4). This is not altogether surprising, as the organisations involved and their procedures will likely be assumed to form part of accepted practice. It is also important to be aware of the tensions and synergies that exist between different purposes of monitoring (Spearman and McGray 2011). In this regard, the proposed framework stresses the importance of a conscious and explicit design of a monitoring and evaluation programme.
